# Prevalence of tobacco use in healthcare workers: A systematic review and meta-analysis

**DOI:** 10.1371/journal.pone.0220168

**Published:** 2019-07-25

**Authors:** Kapka Nilan, Tricia M. McKeever, Ann McNeill, Martin Raw, Rachael L. Murray

**Affiliations:** 1 UK Centre for Tobacco and Alcohol Studies, School of Medicine, Clinical Sciences Building, Nottingham City Hospital, University of Nottingham, Nottingham, United Kingdom; 2 UK Centre for Tobacco and Alcohol Studies, Institute of Psychiatry, Psychology & Neuroscience (IoPPN), King’s College London, London, United Kingdom; 3 NYU College of Global Public Health, New York University, New York, New York, United States of America; 4 NYU Medical School, New York University, New York, New York, United States of America; University of Calfornia San Francisco, UNITED STATES

## Abstract

**Objectives:**

To estimate tobacco use prevalence in healthcare workers (HCW) by country income level, occupation and sex, and compare the estimates with the prevalence in the general population.

**Methods:**

We systematically searched five databases; Medline, EMBASE, CINHAL Plus, CAB Abstracts, and LILACS for original studies published between 2000 and March 2016 without language restriction. All primary studies that reported tobacco use in any category of HCW were included. Study extraction and quality assessment were conducted independently by three reviewers, using a standardised data extraction and quality appraisal form. We performed random effect meta-analyses to obtain prevalence estimates by World Bank (WB) country income level, sex, and occupation. Data on prevalence of tobacco use in the general population were obtained from the World Health Organisation (WHO) Global Health Observatory website. The review protocol registration number on PROSPERO is CRD42016041231.

**Results:**

229 studies met our inclusion criteria, representing 457,415 HCW and 63 countries: 29 high-income countries (HIC), 21 upper-middle-income countries (UMIC), and 13 lower-middle-and-low-income countries (LMLIC). The overall pooled prevalence of tobacco use in HCW was 21%, 31% in males and 17% in females. Highest estimates were in male doctors in UMIC and LMLIC, 35% and 45%, and female nurses in HIC and UMIC, 21% and 25%. Heterogeneity was high (I^2^ > 90%). Country level comparison suggest that in HIC male HCW tend to have lower prevalence compared with males in the general population while in females the estimates were similar. Male and female HCW in UMIC and LMLIC tend to have similar or higher prevalence rates relative to their counterparts in the general population.

**Conclusions:**

HCW continue to use tobacco at high rates. Tackling HCW tobacco use requires urgent action as they are at the front line for tackling tobacco use in their patients.

## Introduction

As health experts and promoters, healthcare workers (HCW) have an important role to play in curbing the global tobacco epidemic [1]. HCW are well placed to promote smoking cessation and treat tobacco dependence in their patients, following evidence-based tobacco cessation guidelines [2,3]. The WHO Framework Convention on Tobacco Control (FCTC) Article 14 specifically stress the importance of HCW being role models and setting an example by not using tobacco [4]. Currently the prevalence of tobacco use in HCW worldwide is unknown.

Previous reviews of the literature reported historical declines in prevalence of smoking among physicians starting in the 50s and 60s in the US, Australia and New Zealand [5–7], and there is some evidence that prevalence is decreasing in nurses in the US and New Zealand [8–10]. A wider international review of tobacco use in the medical porfession found large variation in smoking prevalence in the late 90s, with rates of below 5% in the US and UK physicians, and above 30% in France, Italy, China [11]. A multi-country review of European GPs’ involvement with provision of smoking cessation reported smoking rates of above 40% among GPs in Buylgaria, Greece andSlovakia in the early 2000s [12]. A recent international review of nurses’attitudes to smoking and smoking cessation (2015) found female nurses’smoking rates to range from as low as 2% in China to as high as 25.8% in Northern Ireland, and above 30% in Italy, Serbia and Spain [13]. Another international review of physicians’ tobacco use and smoking cessation practices between 1987–2010, reported regional variation in smoking, with rates of 37% in Central and Eastern Europe, 29% in Africa, 25% in Central and South America, and 17.5% in Asia [14].

Tobacco use data on other healthcare professionals were generally lacking and there were no recent estimates of prevalence of tobacco use in HCW across occupation and country level.

We aimed, therefore, to systematically review literature published since 2000 that report tobacco use in any HCW population in any country, and provide estimates by country income level, sex, and occupation. The second aim was to compare smoking prevalence in HCW to the prevalence in the general population at country level.

## Methods

### Search strategy and selection criteria

We conducted a systematic review and meta-analysis following PRISMA and GATHER guidelines [15,16]. All primary studies from any country and in any language that reported tobacco use in health care populations directly involved in the delivery of health care services to patients (physicians, nurses, dentists, pharmacists, and allied health workers) were eligible for inclusion in the review. First, we searched five electronic databases (MEDLINE, EMBASE, CINHAL Plus, CAB Abstracts, and LILACS) and the WHO Global Health Observatory data repository. A search strategy was developed for each database with a combination of free text and controlled vocabulary (MeSH terms). Search terms included ‘physician’, ‘general practitioner’, ‘medical doctor’, ‘nurse’, ‘pharmacist’, ‘dentist’, combined with terms for tobacco use and tobacco, such as ‘smoking’, ‘tobacco’, ‘cigar*, ‘shisha’, ‘water pipe’, ‘smokeless tobacco’, adapted for each database. An example of the search strategy used for MEDLINE is available in S2 Appendix. Second, reference lists of publications retrieved in the first step and relevant review papers were screened for relevant studies. The latest search was run on 14 March 2016. Title and abstract screening were performed by two researchers (KN, TM) independently and any conflicts resolved by discussion. If agreement were not reached, a third reviewer was consulted (RM); duplicates and records that did not meet eligibility criteria were excluded at this stage. The full text of potentially eligible abstracts was retrieved, and those that could not be accessed or translated were excluded. Conference abstracts that contained relevant data were included.

### Study selection, data extraction and quality assessment

Three researchers (KN, RM, TM) assessed study quality and extracted relevant data independently in pairs, using a standardised data extraction and quality assessment criteria form. Studies were assessed against nine criteria, worth one point each, equally weighted and included selection and representativeness of the study participants, response rate, reporting and measuring tobacco use, research finding and conflict of interest. Data and study scores were reviewed and disagreements resolved by discussion.

We extracted data on number of participants in the study, sex, occupation, and prevalence of tobacco use. We used current, former and never smoker categories; occasional users were allocated to the current smoker category. We used the terms ‘tobacco use’ to cover any tobacco use (cigarettes, water pipe, smokeless tobacco). Studies that reported combined prevalence for more than one occupational category, or unspecified HCW or mixed hospital personnel, were assigned to a ‘mixed’ category. Studies including healthcare professionals who were not medical doctors, dentists, nurses or pharmacists were assigned to an ‘allied’ category. Studies with prevalence data for two or more defined HCW categories, year or country, were extracted multiple times. When there were multiple studies with prevalence data from the same cohort, we included only one study per cohort, selected on the basis of the study sample size and its overall quality. For studies that did not specify the sample size we used the median sample size calculated from all studies (n = 375). Where studies did not specify the year of data collection, we assigned the year before the study was published as the year of data collection. Full text studies that contained prevalence data prior to 2000 were excluded from the meta-analyses.

### Data analysis

We grouped the studies by country income level according to the WB income classification for July 2016 fiscal year: high-income countries (HIC), upper-middle income countries (UMIC), lower middle-income countries (LMIC) and low-income counties (LIC). Due to a low number of studies from LMIC and LIC we combined them under lower middle-income and low-income countries (LMLIC) category. We conducted random effect meta-analyses to obtain pooled prevalence estimates by WB income level with 95% Confidence Interval (CI). Heterogeneity between studies was assessed using the I^2^ statistic. We performed subgroup analyses by HCW occupation and sex, and sensitivity analyses by quality score (grouping studies below and above the mean score) and year of data collection, to explore the effects of heterogeneity. All analyses were conducted in Stata 14.

In the second step of the analyses, we estimated the mean prevalence in male and female HCW by country. WHO Global Health Observatory tobacco use prevalence data in adult males and females for 2015 were used to for country level comparison.

## Results

### Study selection

**Fig 1:**
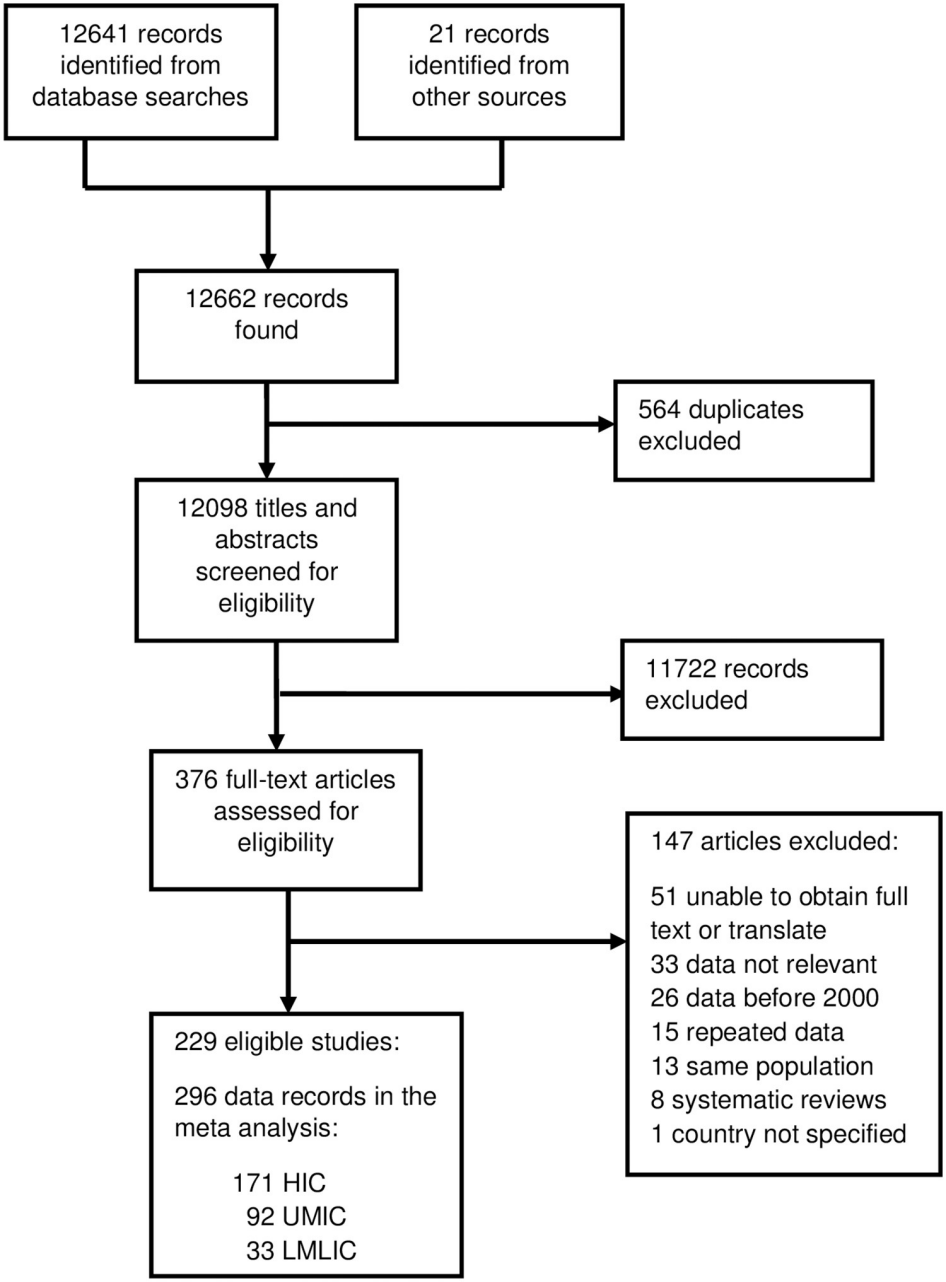
Study selection.

We identified 12662 records, 229 of which met our inclusion criteria [17–245]. 55 of the selected studies contained prevalence data on more than one HCW category or year, hence the total number of data records included in the meta-analyses was 296 (171 from 29 HIC, 92 from 21 UMIC and 33 from 13 LMLIC) with prevalence data on 457,415 HCW collected between 2000 and 2014 (Fig 1).

HCW occupation categories included in studies were medical doctors (n = 122), nurses (n = 65), allied (n = 16), dental (n = 14), pharmacy (n = 8), and mixed (n = 71). Most studies reported cigarette smoking (n = 235), three studies reported water pipe smoking [72,117,118] and three smokeless tobacco [42,96,228].

Study sample sizes ranged from 32 to 102,635, with a median sample size of 375. The mean quality score was 4.2 (SD 1.6) out of a possible maximum of 9; quality scores ranged from one to eight and were on average highest in HIC (4.4, SD 1.8) and lowest in UMIC (3.78, SD 1.40). Overall, most studies had a clearly defined study population (87%), stated the study period (77%) and used anonymous survey methods (69%); however, the majority of studies lost points on quality criteria assessing participant selection (only 32% had a representative sample and 40% used a random sampling approach), participant response rate (only 36% had a response rate of 70% or above), and quality of tobacco use data (only 38% clearly defined and measured tobacco use) (S3 Appendix) The studies main characteristics, quality score and reported prevalence of tobacco use are summarised in Tables A—C in S4 Appendix.

### Prevalence of tobacco use in HCW by country income level

We estimated the pooled prevalence of tobacco use among HCW by grouping studies by WB country income level. The overall pooled prevalence was 21% (95% CI 20–23) and varied by WB income level (p = 0·01). The highest pooled prevalence was in UMIC at 25% (95% CI 22–28) and lowest in LMLIC at 19% (95% CI 15–23) (Table 1).

**Table 1:** Pooled prevalence of tobacco use in HCW by WB country income level.

Year of data collection (2000–2014)	Countries by income level				P-value for difference across income level
	All	HIC	UMIC	LMLIC	
**Pooled prevalence, % (95% CI)**	21 (20–23)	20 (18–21)	25 (22–28)	19 (15–23)	0·01
**Studies (n)**	296	171	92	33	
**Countries (n)**	67	33	21	13	
**I**^**2**^**%**	99·5	99·6	99·2	97·1	99·5

HCW = healthcare workers; WB = World Bank; HIC = high income countries; UMIC = upper middle income countries LMLIC = lower middle and low income countries.

### Prevalence of tobacco use in HCW by country income level and year of data collection

We estimated the pooled prevalence of tobacco use among HCW by grouping studies by country income level and year of data collection (Figures A-E in S6 Appendix). Overall, the estimates suggest some decreases in prevalence in HIC and UMIC, while there was no significant change in prevalence in LMLIC. The overall prevalence for 2011–2015 did not vary significantly by country income level (p = 0·94) (Table 2).

**Table 2:** Pooled prevalence of tobacco use in HCW by country income level and year of data collection.

Years of data collection	Countries by income level				P-value for difference across income level
	All	HIC	UMIC	LMLIC	
**2000–2005**					
**Pooled prevalence, % (95% CI)**	23 (21–25)	22 (20–24)	27 (22–32)	14 (8–20)	0·01
**Studies (n)**	120	82	31	7	
**Countries (n)**	40	22	12	6	
**I**^**2**^**%**	99·6	99·7	99·2	95·9	
**2006–2010**					
**Pooled prevalence, % (95% CI)**	22 (20–24)	17 (15–19)	27 (22–31)	22 (16–29)	<0·01
**Studies (n)**	104	50	39	15	
**Countries (n)**	38	16	15	7	
**I**^**2**^**%**	99·3	98·9	99·2	97·7	
**2011–2015**					
**Pooled prevalence, % (95% CI)**	18 (16–21)	19 (15–22)	19 (14–23)	18 (12–23)	0·94
**Studies (n)**	72	39	22	11	
**Countries (n)**	33	19	8	6	
**I**^**2**^**%**	99·3	99·5	97·8	95·8	
**P- value for difference across years**	0·04	0·01	0·01	0.22	0·94
**I**^**2**^**%**	99·5	99·6	99·2	97·1	

HCW = healthcare workers; WB = World Bank; HIC = high income countries; UMIC = upper middle income countries LMLIC = lower middle and low income countries.

### Prevalence of tobacco use in HCW by sex

We estimated prevalence by sex from 78 studies that reported tobacco use in male HCW and 97 in female HCW (Figures F and G in S6 Appendix). The pooled prevalence was almost twice as high in males than females 31% (95% CI 28–34) vs 17% (95% CI 15–18) and varied by income level (Table 3). Male HCW in UMIC and LMLIC had a higher prevalence compared with their counterparts in HIC, 37% (95% CI 34–40) and 37% (95% CI 21–53) vs 24% (95% CI 21–27) (p<0·01). The pooled prevalence in female HCW was higher in HIC and UMIC compared with their counterparts in LMLIC, 19% (95% CI 16–21) and 18% (95% CI 15–20) respectively vs 4% (95% CI 2–6) (p<0·01).

**Table 3:** Pooled prevalence of tobacco use in HCW by WB country income level and sex.

Countries by income level	All	HIC	UMIC	LMLIC	P-value for difference across income level
**Male HCW**					
**Pooled prevalence, % (95% CI)**	31 (28–34)	24 (21–27)	37 (34–40)	37 (21–53)	<0·01
**Studies (n)**	78	40	27	11	
**Countries (n)**	36	21	10	5	
**I**^**2**^**%**	99·4	99	99·1	98·7	
**Female HCW**					
**Pooled prevalence, % (95% CI)**	17 (15–18)	19 (16–21)	18 (15–20)	4 (2–6)	<0·01
**Studies (n)**	97	47	38	12	
**Countries (n)**	46	25	15	6	
**I**^**2**^**%**	94·3	99·7	99·2	92·5	

HCW = healthcare workers; WB = World Bank; HIC = high income countries; UMIC = upper middle income countries LMLIC = lower middle and low income countries.

### Prevalence of tobacco use in HCW by occupation

We estimated prevalence by occupation and country income level for medical doctors, nurses, dental, pharmacy, allied, and mixed HCW (Figures H–O in S6 Appendix). Overall, nurses and mixed hospital personnel had the highest pooled prevalence at 24% (95% CI 22–26) and 24% (95% CI 21–28) respectively, while pharmacy HCW had the lowest at 14% (95% CI 8–20). Country income level differences in prevalence were statistically significant in medical, nursing, dental and pharmacy HCW, with the highest estimates in UMIC (p<0·01) (Table 4).

**Table 4:** Pooled prevalence of tobacco use in HCW by WB country income level and occupation.

HCW occupation	Countries by income level				P-value for difference across income level
	All	HIC	UMIC	LMLIC	
**Medical**					
**Pooled prevalence, % (95% CI)**	20 (19–22)	16 (14–18)	25 (22–28)	21 (16–26)	<0·01
**Studies (n)**	122	62	44	16	
**Countries (n)**	48	23	15	10	
**I**^**2**^**%**	99·1	99.1	97·7	97·2	
**Nursing**					
**Pooled prevalence, % (95% CI)**	24 (22–26)	24 (21–26)	27 (17–37)	7 (4–13)	<0·01
**Studies (n)**	65	49	15	1	
**Countries (n)**	32	21	10	1	
**I**^**2**^**%**	99·7	99·7	99·4	-	
**Dental**					
**Pooled prevalence, % (95% CI)**	18 (12–23)	19 (8–29)	21 (12–30)	8 (6–10)	<0·01
**Studies (n)**	14	7	5	2	
**Countries (n)**	10	5	4	1	
**I**^**2**^**%**	97·1	97·9	96·3	-	
**Pharmacy**					
**Pooled prevalence, % (95% CI)**	14 (8–20)	11 (5–17)	39 (29–49)	-	<0·01
**Studies (n)**	8	7	1	-	
**Countries (n)**	5	4	1	-	
**I**^**2**^**%**	97.2	97·2	-	-	
**Allied**					
**Pooled prevalence, % (95% CI)**	15 (11–20)	14 (9–19)	17 (13–20)	19 (3–35)	0·65
**Studies (n)**	16	11	2	3	
**Countries (n)**	10	6	2	2	
**I**^**2**^**%**	97·4	97·9	0	0	
**Mixed**					
**Pooled prevalence, % (95% CI)**	24 (21–28)	26 (21–31)	25 (19–31)	19 (13–26)	0·32
**Studies (n)**	71	35	25	11	
**Countries (n)**	32	15	11	6	
**I**^**2**^**%**	99·5	99·8	98·5	97·8	

HCW = healthcare workers; WB = World Bank; HIC = high income countries; UMIC = upper middle income countries LMLIC = lower middle and low income countries.

### Prevalence of tobacco use in medical doctors and nurses by sex and country income level

We estimated prevalence of tobacco use by sex for the two occupational categories for which most data were available—medical doctors and nurses (Figures P–S in S6 Appendix). Overall, male medical doctors had a higher pooled prevalence than their female counterparts, 29% (95% CI 25–32) vs. 12% (95% CI 10–13) (Table 5). Male doctors in UMIC and LMLIC had higher estimates compared with their counterparts in HIC, at 35% (95% CI 32–39) and 45% (95% CI 26–64) respectively vs 19% (95% CI 15–22), and the difference was statistically significant (p <0·01). In female doctors prevalence estimates did not vary by income level (p = 0·82).

**Table 5:** Pooled prevalence of tobacco use in medical doctors and nurses by WB country income level and sex.

HCW occupation	Countries by income level				P-value for difference across income level
	All	HIC	UMIC	LMLIC	
**Medical doctors (males)**					
**Pooled prevalence, % (95% CI)**	29 (25–32)	19 (15–22)	35 (32–39)	45 (26–64)	<0·01
**Studies (n)**	45	21	20	4	
**Countries (n)**	28	16	9	3	
**I**^**2**^**%**	99·5	99.4	92·1	95·1	
**Medical doctors (females)**					
**Pooled prevalence, % (95% CI)**	12 (10–13)	12 (9–14)	13 (11–16)	10 (1–20)	<0·82
**Studies (n)**	43	20	19	4	
**Countries (n)**	27	15	9	3	
**I**^**2**^**%**	98·6	98·3	99·1	91·2	
**Nurses (males)**					
**Pooled prevalence, % (95% CI)**	28 (18–37)	29 (26–32)	47 (37–57)	0 (0–66)	<0·01
**Studies (n)**	10	8	1	1	
**Countries (n)**	6	4	1	1	
**I**^**2**^**%**	94·3	25·1	-	-	
**Nurses (females)**					
**Pooled prevalence, % (95% CI)**	22 (18–25)	21 (16–25)	24 (18–30)	7 (3–13)	<0·01
**Studies (n)**	30	16	13	1	
**Countries (n)**	22	11	10	1	
**I**^**2**^**%**	99.9	99·9	99	-	

HCW = healthcare workers; WB = World Bank; HIC = high income countries; UMIC = upper middle income countries LMLIC = lower middle and low income countries.

Similar to doctors, male nurses had a higher pooled prevalence than female nurses, 28% (95% CI 18–37) vs. 18% (95% CI 14–22) and varied by income level (p <0·01) and were significantly higher in male nurses in UMIC and LMLIC (based on 1 study each), at 47% (95% CI 37–57) and 0% (95% CI 0–66) compared with their counterparts in HIC (p <0·01). In female nurses the prevalence was significantly higher in UMIC and HIC at 24% (95% CI 18–30) and 21% (95% CI 16–25) respectively compared with their counterparts in LMLIC, at 7% (95% CI 3–13) (based on one study) (p <0·01).

Overall, female nurses seemed to have higher prevalence estimates compared with female medical doctors (18% vs 12%). This was observed in female nurses in HIC and UMIC (21% vs 12% and 24% vs 13% respectively), while in LMLIC the pattern was reversed (7% vs 10%).

Male nurses in HIC and UMIC also seemed to have higher prevalence estimates than male doctors (29% vs 19% and 47% vs 35% respectively). In LMLIC prevalence estimates were higher in male doctors than male nurses (45% vs 0% respectively), although only one study reported data on male nurses in this group.

### Country level comparison of prevalence of tobacco use in HCW and the general population

#### Male HCW compared with males in the general population in HIC

In most HIC, the mean prevalence in male HCW tend to be lower compared with their counterparts in the general population (between two and 22.5 percentage points) (Table A in S5 Appendix). The exceptions were Australia, Italy and Uruguay, where the prevalence was higher in HCW. The lowest prevalence (<5%) was in the US and Ireland, and the highest (> 30%) in Greece, Croatia, Italy and Uruguay. A number of countries (Czech Republic, Denmark, Finland, Germany, Korea, Saudi Arabia, and the UK) were lacking prevalence data on HCW.

#### Male HCW compared with males in the general population in UMIC

The mean prevalence in male HCW in UMIC was above 20%. In Russia, China, Bosnia and Herzegovina, the prevalence in HCW was lower compared with males in the general population (between 0.1 and 26 percentage points), and in Mexico, Turkey, Brazil, Iran, Ecuador the prevalence was higher in HCW (between 2.9 and 21 percentage points). The lowest prevalence (<30%) was in Argentina, Brazil and Mexico and the highest (>40%) in Turkey. Countries lacking prevalence data on male HCW included Colombia, Cuba, Dominican Republic, Jamaica, Jordan, Malaysia, Peru, Romania, Serbia, South Africa, Venezuela.

#### Male HCW compared with males in the general population in LMLIC

In LMLIC, except Pakistan, the mean prevalence in male HCW was lower than in males in the general population (between 13 and 54 percentage points). The lowest prevalence (<10%) was in India and the highest (>50%) in Pakistan. Countries with prevalence higher than 20% in HCW included Indonesia and Nepal, higher than 30% Armenia and Syria, and above> 50% Pakistan and Tunisia.

#### Female HCW compared with females in the general population in HIC

In a number of HIC the mean prevalence in female HCW was lower (between 0.7 and 15 percentage points) compared with their counterparts in the general population while in another groups of countries the prevalence was higher in HCW (between 1 and 19 percentage points) (Table B in S5 Appendix). The lowest prevalence was in Korea, Oman and Qatar, less than 5%. HCW prevalence was higher than 20% in Chile, Czech Republic, Denmark, France, Italy, higher than 30% in Croatia, Spain, Uruguay, and above 40% in Greece. Countries with no prevalence data on female HCW included Germany, Bahrain, Finland, Saudi Arabia and the UK.

#### Female HCW compared with females in the general population in UMIC

In the majority of UMIC (except for Jamaica and Lebanon), the mean prevalence in female HCW was higher than in females in the general population. The lowest prevalence was in Iran and Jamaica, less than 5%. The prevalence in HCW was higher than 20% in Ecuador, Jordan and Turkey, higher than 30% in Cuba and Argentina, and above 40% in Bosnia and Herzegovina.

#### Female HCW compared with females in the general population in LMLIC

Female prevalence data on female HCW from LMLIC were limited. HCW in Armenia and Pakistan had a higher prevalence than female in the general population, by 13 and 7 percentage points, while female HCW in India, Indonesia and Nepal had a lower prevalence. The lowest prevalence was in Indonesia, Nepal and Syria (<5%) and highest in Armenia and Pakistan (<15%).

## Discussion

This study estimated that between 2000 and 2014 21% of HCW were tobacco users, and there was significant variation in prevalence by sex, occupation and country income level. The income differences in prevalence between countries were present in almost all occupations but were most pronounced in male doctors and female nurses. Male medical doctors in UMIC and LMLIC and female nurses in HIC and UMIC had the highest prevalence estimates. Male and female nurses in HIC and UMIC had higher estimates than their medical doctor counterparts, which suggests socioeconomic differences at play in addition to sex and income differences.

Compared with the smoking prevalence rates in the general population of their countries, male HCW in high income countries tend to have lower prevalence rates while male and female HCW in middle and low income countries tend to have similar or higher prevalence rates relative to their counterparts in the general population.

To our knowledge, this is the first systematic review that attempts to provide estimates of the prevalence of tobacco use worldwide across a variety of different HCW. The high prevalence in male medical doctors in UMIC and LMLIC compared with HIC confirms the findings of previous reviews [11,14]. The sex and income differences in tobacco use among HCW appear to reflect the global pattern of the tobacco epidemic in the general population, which is spreading from high income to low and middle income countries, and is beginning and declining earlier in males than females [246,247]. Overall, the highest prevalence and the largest sex differences in prevalence were observed in middle and low income countries, which are at earlier stages of the tobacco epidemic. By contrast, both male and female medical doctors in HIC, most of which are at the later stages of the epidemic, have the lowest prevalence.

While female nurses had a higher prevalence compared to female doctors in HIC and UMIC, the opposite pattern was found in middle and low income countries where female doctors had a higher prevalence compared to female nurses. A positive association between tobacco use and a higher socioeconomic status was found previously among professional women in LMLIC [248,249]. Tobacco industry’s targeting of women in LMICs who have lower rates of smoking than men particularly in Asia, Sub-Saharan Africa and the Middle East, is similar to the industry’s targeting of women in HIC in the 1920s, linking smoking to women’s social and economic freedoms [250].

High prevalence of smoking among hospital personnel suggests high levels of second-hand smoke exposure in hospitals. Smoking among hospital healthcare workers is a major barrier to implementation of smokefree policy in hospitals. Some hospital studies also reported non-compliance with smoking restrictions on hospital premises [25,181] and smoking in front of patients [25,196]. Second-hand smoke is a health risk and there are no safe levels of exposure [251]. Smokefree workplace can encourage smokers to quit [252]. Although the majority of hospital personnel who smoked wanted to quit, there was little knowledge or cessation support available [85,99,121,192].

Country level comparisons showed that male HCW in HIC were the only group that had a lower prevalence compared with their counterparts in the general population, while in the middle and low income countries prevalence rates in both male and female HCW were similar or higher compared with their counterparts in the general population. Male doctors and female nurses in middle income countries appear to have some of the highest prevalence rates.

Despite some limitations of data (WHO prevalence data were more recent than HCW data), these finding seems to confirm the hypothesis that similarly to high income countries, the smoking epidemic in low and middle income countries starts in more affluent socioeconomic groups, e.g. health professionals, before it spreads to the rest of the population, and begins to decline in males before females in the later stages of the epidemic.

These findings offer cause for concern as many middle and low income countries have fewer tobacco control measures in place [253] and inadequate provision of cessation support [254] There have already been increases in smoking prevalence in males in the general population in some of these countries [253,255], and any further increases are likely to be observed in HCW populations as well.

Despite an increasing number of countries introducing tobacco control legislation as part of the WHO FCTC, progress in implementation of tobacco control measures has been slow particularly in low and middle income countries [254,256], with an overall decrease in smoking prevalence in the general population globally of 1.1 percentage point between 2010 and 2015 [253]. Offering support to HCW to stop using tobacco is a key recommendation of the FCTC Article 14 guidelines, however according to a recent survey of 142 countries, only 44% of countries offer help to HCW to stop using tobacco [254].

A number of limitations need to be considered. We excluded 51 potentially eligible studies because we were unable to access the full text or translate them. Studies were not evenly distributed across income groups or occupation. More than half of all studies were from HIC, compared to just 12% from LMLIC. Over half of occupations represented were medical doctors, whereas dentists, pharmacists and allied HCW were represented in less than 10% of studies. Further, the majority of studies were of poor quality due to small sample sizes, low response rates and tobacco use being not clearly defined and measured. Despite the large number of studies included in the review, only 33% of studies had nationally representative samples; mainly due to studies reporting on data collected in just one hospital. There are relatively few recent studies, the most recent reporting prevalence data from 2014, and in a number of categories there were a lack of data reported. There was a high heterogeneity (above 90%) in almost all meta-analyses, including sub-group analyses, which suggests high variability in study populations and approaches.

Individual categories of HCW were not homogenous, for example studies included in the medical doctor category included professional groups such as cardiology [73,81,136] and respiratory physicians, [115,116,170] who may reasonably be expected to have a lower tobacco use prevalence than other specialties which have a less clear link with smoking, thus potentially biasing the results.

The overall prevalence estimates in meta-analyses based on mixed female and male populations in countries with large sex difference in prevalence should be interpreted with caution. The occupational differences in smoking prevalence between doctors and nurses in countries with a large proportion of nursing workforce being female are likely to be biased by the gender differences in prevalence, especially in countries like China where smoking prevalence is considerably lower in females compared with males.

HCW data were generally older, and for some countries more than a decade older than the WHO population data. It is possible, therefore, that the prevalence in HCW in low and middle income countries is underestimated as it must have increased since then and be similar to that in the general population.

In all studies tobacco use was self-reported; 66% used self-administered anonymous questionnaires to verify smoking status. While self-reported smoking status was shown to be accurate in most observational studies [257] under-reporting of smoking in female participants due to cultural or social norms in some countries [74] or in countries with a growing popularity in water pipe smoking as this form of tobacco use is not perceived as harmful or addictive [258] is a concern and results should be considered accordingly.

Despite wide variations in prevalence of tobacco use among HCW by country income level, gender and occupation, there are no evidence of significant recent declines in prevalence of tobacco use. In middle and low income countries the prevalence of tobacco use among HCW is no lower than that in the general population and in some professions is very high indeed. This presents a particular challenge for countries where smoking prevalence is already high and/or is projected to increase. This is a cause for concern due to the critical role HCW play in advising tobacco users to stop.

While smoking prevalence in HIC has been associated with social and economic disadvantage, smoking among female HCW in LMLIC may be an indicator of the rise of the tobacco epidemic in these countries, and as such should be addressed by interventions targeting specifically women [249].

The Article 14 Guidelines of the WHO FCTC (4) recommends that HCW record patients’ tobacco use and provide at least brief smoking cessation advice. Health professionals who use tobacco maybe less likely to fulfil this obligation or even if they did, their own tobacco dependence would undermine the authority and credibility of cessation advice. All HCW have a duty to provide smoking cessation advice to patients. System changes should be implemented to encourage HCW engagement in smoking cessation regardless of their own smoking status.

Offering support to HCW to stop using tobacco is a priority recommendation of the FCTC Article 14 guidelines. Workplace “smoking breaks” are not statutory right and should be discouraged as a potential disincentive for quitting smoking. Equally, lack of cessation support for HCW who smoke can seriously undermine the success of introducing smokefree policy in healthcare settings [259]. Stopping smoking should be encouraged and staff should be allowed to attend smoking cessation services during working hours. Smokefree work places can encourage changes in smoking behaviour [252]. Studies assessing the impact of implementation of smokefree policy in healthcare settings have shown that smoking bans combined with cessation support are effective in decreasing smoking prevalence in both patients’ and staff [260,261].

Tobacco treatment and preventive measures specifically targeting HCW should be a priority in all countries. Such measures may include work place interventions, restriction on smoking while at work and provision of cessation support for staff who smoke.

## Conclusions

Tobacco use in HCW is a seriously neglected area. HCW are key in reducing tobacco use in the population in their position as role models, and the high levels of tobacco use amongst these groups especially in middle- and low-income countries is concerning and suggests a need for targeted action to address this.

The systematic review also highlighted that the monitoring of HCW tobacco use has been neglected; a number of occupational groups were lacking in data and in many cases data which were available were from older, low quality studies. Global monitoring of tobacco use among HCW is urgently needed.

## Supporting information

S1 Appendix PRISMA checklist 2009. (DOC)

S2 Appendix Example search strategy. (DOC)

S3 Appendix Summary of quality score. (DOC)

S4 Appendix Summary of studies. (DOC)

S5 Appendix Country level comparison of prevalence of tobacco use in HCW and the general population. (DOCX)

S6 Appendix Meta-analyses. (DOC)
