# Supplementary material for: Prevalence of tobacco use in healthcare workers: A systematic review and meta-analysis
Source: PLoS One. 2019 Jul 25;14(7):e0220168. doi: 10.1371/journal.pone.0220168 (PMC6657871; doi:10.1371/journal.pone.0220168)
Supplement: S2 Appendix — (DOC) [file pone.0220168.s002.doc]

# S2 Appendix. Example search strategy.

**Medline database search terms**

1. exp Students, Health Occupations/
2. exp Health Personnel/
3. exp Community Health Workers/
4. exp Mental Health Services/
5. exp Emergency service,
6. hospital/
7. exp Emergency Medical Services/
8. physician*
9. "general practitioner*"
10. "family doctor"
11. "medical doctor"
12. "clinical officer"
13. "mental health personnel"
14. nurse*
15. pharmacist*
16. dentist*
17. exp Pharmacists
18. exp Dentists/
19. any of 1 – 18
20. exp "Tobacco Use"/
21. exp Smoking/
22. exp Tobacco
23. Smokeless/
24. smoking.mp.
25. "smoking behaviour".mp.
26. "smoking status".mp.
27. "smoking prevalence".mp.
28. "tobacco use".mp.
29. shisha.mp.
30. waterpipe.mp.
31. "water pipe".mp.
32. cigar*.mp.
33. "smokeless tobacco".mp.
34. any of 20 – 33
35. 19 and 34
36. Limit 35 to (humans and last 16 years)
