# Supplementary material for: Prevalence of tobacco use in healthcare workers: A systematic review and meta-analysis
Source: PLoS One. 2019 Jul 25;14(7):e0220168. doi: 10.1371/journal.pone.0220168 (PMC6657871; doi:10.1371/journal.pone.0220168)
Supplement: S3 Appendix — (DOC) [file pone.0220168.s003.doc]

# S3 Appendix. Summary of quality score.

| **Quality score criterion** | **Overall**  **%** | **HIC**  **%** | **UMIC**  **%** | **LMLIC**  **%** |
| --- | --- | --- | --- | --- |
| Study population clearly defined  (*Categories of HCW clearly stated)* | 87 | 86 | 87 | 91 |
| Representative sample  (*A clear sampling frame; participants drawn from more than one setting)* | 32 | 36 | 29 | 21 |
| Response rate >70%  (*The number of valid responses as a % of all participants in the survey*) | 36 | 29 | 40 | 55 |
| Study period defined  (*The year/s of data collection clearly stated*) | 77 | 76 | 79 | 85 |
| Tobacco use clearly defined and measured  (*Type of tobacco defined, current/former/never user defined)* | 38 | 41 | 36 | 36 |
| Random selection of participants  (*Probability sampling approach used)* | 40 | 46 | 34 | 30 |
| Self-administered anonymous survey  (*An anonymous data collection method used rather than face to face, interview etc*) | 69 | 78 | 55 | 70 |
| Biochemical measurements  (*Smoking status biochemically verified e.g. measuring CO or cotinine*) | 0.9 | 0 | 1 | 1 |
| Research funding and conflict of interest  (*Declaration of conflict of interest and funding included*) | 22 | 27 | 10 | 18 |
