# Supplementary material for: Prevalence of tobacco use in healthcare workers: A systematic review and meta-analysis
Source: PLoS One. 2019 Jul 25;14(7):e0220168. doi: 10.1371/journal.pone.0220168 (PMC6657871; doi:10.1371/journal.pone.0220168)
Supplement: S4 Appendix — (DOC) [file pone.0220168.s004.doc]

# S4 Appendix. Summary of studies.

**Table A. Summary of studies in lower-middle and low income countries (LMLIC)**

| **Author** | **Country** | **Year data collected** | **HCW Category** | **Type of tobacco use** | **Study quality score** | **Sample size** | | **Tobacco use (%)** |
| --- | --- | --- | --- | --- | --- | --- | --- | --- |
| Adraneda(19) | Philippines | 2014 | Medical | Cigarettes | 3 | 557 | | 27·8 |
| Ammar(25) | Tunisia | 2010 | Mixed | Cigarettes | 4 | 570 | | 24·8 |
| Bansal(33) | India | 2011 | Medical | Cigarettes | 2 | 202 | | 22 |
| Chandrashekar(51) | India | 2006 | Dental | Cigarettes | 5 | 114 | | 4·4 |
| Chaudhry(53) | Pakistan | 2006 | Mixed | Cigarettes | 5 | 300 | | 28 |
| Fakhfakh(74) | Tunisia | 2005 | Mixed | Cigarettes | 5 | 796 | | 9·4 (F) |
| Ketkar(110) | India | 2012 | Mixed | Cigarettes | 3 | 247 | | 7 |
| Kumar(117) | Pakistan | 2011 | Medical | Water pipe | 3 | 343 | | 29·5 |
| Kumar(118) | Pakistan | 2014 | Medical | Cigarettes | 3 | 786 | | 16·3 |
| Malik(130) | Pakistan | 2009 | Medical  Allied | Cigarettes | 6 | 234  207 | 37·2  35·7 | |
| Memon(143) | Pakistan | 2008 | Medical  Allied | Cigarettes | 4 | 45 | 22·2  11·8 | |
| Mitra(147) | India | 2013 | Dental | Cigarettes | 4 | 500 | 10 | |
| Movsisyan(150) | Armenia | 2009 | Medical  Nursing | Cigarettes | 5 | 93  112 | 37·6  7 | |
| Nawaz(153) | Pakistan | 2006 | Medical | Cigarettes | 4 | 227 | 36 | |
| Ng(156) | Indonesia | 2003 | Medical | Cigarettes | 5 | 447 | 11·6 | |
| Ngahane(157) | Cameroon | 2010 | Medical | Cigarettes | 3.5 | 192 | 3·6 | |
| Nollen(158) | Nigeria | 2002 | Medical | Cigarettes | 3 | 373 | | 3 |
| Pati(168) | India | 2013 | Medical | Cigarettes | 3 | 91 | | 13·2 |
| Paudel(169) | Nepal | 2012 | Mixed | Cigarettes | 2 | 500 | | 6 |
| Perrin(173) | Armenia | 2004 | Medical | Cigarettes | 5.5 | 238 | | 34 |
| Pokhrel(175) | Nepal | 2005 | Mixed | Not specified | 5 | 377 | | 20·4 |
| Salah(196) | Tunisia | 2008 | Mixed | Cigarettes | 4 | 452 | | 19 |
| Shelley(205) | Vietnam | 2012 | Mixed | Cigarettes | 5.5 | 114 | | 9·7 |
| Touré(222) | Senegal | 2004 | Medical  Allied | Cigarettes | 4 | 96  153 | | 13·5  10·5 |
| Touré(223) | Senegal | 2007 | Mixed | Cigarettes | 4.5 | 662 | | 12·8 |
| Valliani(228) | Pakistan | 2009 | Mixed | Smokeless tobacco | 6 | 560 | | 48·6 |
| Vanphanom(230) | Laos | 2007 | Medical | Cigarettes | 6 | 855 | | 9·2 |
| Zafar(237) | Pakistan | 2013 | Mixed | Cigarettes | 4 | 180 | | 29·4 |
| Zakaria(238) | Syria | 2011 | Medical | Cigarettes | 3 | 466 | | 24 |

HCW= healthcare workers; (M)=male; (F)=female.

**Table B. Summary of studies in upper middle income countries (UMIC)**

| **Author** | **Country** | **Year data collected** | **HCW Category** | **Type of tobacco use** | **Study quality score** | **Sample size** | **Tobacco use (%)** |
| --- | --- | --- | --- | --- | --- | --- | --- |
| Abrizah(17) | Malaysia | 2009 | Dental | Cigarettes | 4 | 490 | 12·7 |
| Akvardar(21) | Turkey | 2003 | Medical | Cigarettes | 3 | 153 | 37·9 |
| Amiri(24) | Iran | 2007 | Medical | Cigarettes | 5 | 218 (M) 258 (F) | 37·4 (M) 1·2 (F) |
| An(26) | China | 2012 | Nursing | Cigarettes | 7 | 94 (M)  705 (F) | 46·7 (M) 3·4 (F) |
| Aydin(29) | Turkey | 2012 | Medical | Cigarettes | 1 | 678 (M) 507 (F) | 31·6 (M) 37·3 (F) |
| Balbani(31) | Brazil | 2005 | Medical | Cigarettes | 5 | 209 | 7·1 |
| Baltaci(32) | Turkey | 2014 | Medical | Cigarettes | 3 | 261 | 29·1 |
| Barbosa(34) | Brazil | 2008 | Mixed | Cigarettes | 7 | 1759 | 15·7 |
| Bourne(44) | Jamaica | 2008 | Mixed | Cigarettes | 5 | 212 | 4·7 |
| Cakir(46) | Turkey | 2005 | Mixed | Cigarettes | 5 | 883 | 34·2 |
| Calgan(47) | Turkey | 2006 | Pharmacy | Cigarettes | 4.5 | 83 | 38·6 |
| Ceraso(49) | China | 2006 | Medical | Cigarettes | 4 | 103 (M) | 49·5 (M) |
| Chan(50) | China | 2003 | Nursing | Cigarettes | 6 | 1690 | 2·2 |
| Custodio(59) | Brazil | 2008 | Nursing | Cigarettes | 3 | 130 | 35·4 |
| Demir(61) | Turkey | 2011 | Medical | Cigarettes | 5 | 263 | 25·4 |
| Demiralay(62) | Turkey | 2001 | Medical | Cigarettes | 6 | 401 | 43·4 |
| Demiralay(63) | Turkey | 2001 | Medical | Cigarettes | 2 | 135 | 33·7 |
| Echer(69) | Brazil | 2008 | Mixed | Cigarettes | 5 | 848 | 11·8 |
| Gigliotti(84) | Argentina | 2011 | Medical | Cigarettes | 4 | 448 | 20·1 |
| Günay(89) | Turkey | 2009 | Medical | Cigarettes | 4 | 116 | 53·4 |
| Gunes(90) | Turkey | 2002 | Medical | Cigarettes | 4 | 257 | 37·4 |
| Han(91) | China | 2012 | Mixed | Cigarettes | 2 | 3787 | 15·5 |
| He(92) | China | 2011 | Medical | Cigarettes | 5 | 876 | 24·2 |
| Hidalgo(94) | Colombia | 2011 | Medical | Cigarettes | 3 | 38 | 50 |
| Hodgetts(97) | Bosnia & Herzegovina | 2002 | Mixed Medical Nursing | Cigarettes | 5.5 | 205 110 95 | 45 40 51 |
| Jardim(100) | Brazil | 2008 | Medical | Cigarettes | 4 | 72 | 5·6 |
| Jardim(101) | Brazil | 2013 | Mixed | Cigarettes | 5 | 215 | 3·7 |
| Jiang(102) | China | 2004 | Medical | Cigarettes | 5.5 | 1958 (M) 1594 (F) | 41(M) 1(F) |
| Karahan(106) | Turkey | 2005 | Mixed | Cigarettes | 2.5 | 1600 | 37·4 |
| Kheradmand(112) | Iran | 2014 | Mixed | Cigarettes | 3 | 524 | 21·2 |
| Lazovic(121) | Serbia | 2006 | Mixed | Cigarettes | 3 | 462 | 47·2 |
| Li(122) | China | 2005 | Medical | Cigarettes | 3 | 267 | 36·8 |
| Li(123) | China | 2009 | Medical | Cigarettes | 3 | 432 (M) 568 (F) | 37·5 (M) 0·4(F) |
| Li(124) | China | 2011 | Medical | Cigarettes | 5 | 805 (M) 768 (F) | 27·9 (M) 0·5(F) |
| Lindo(126) | Jamaica | 2003 | Medical Nursing Mixed | Cigarettes | 5 | 99 113 212 | 4·2 3·5 5·1 |
| Lisanti(127) | Argentina | 2012 2004 | Mixed | Cigarettes | 2 | 263 | 26·7 36·3 |
| Lopez-Maya(128) | Mexico | 2011 | Medical | Cigarettes | 4 | 258 | 21·7 |
| Man(132) | Romania | 2009 | Medical | Cigarettes | 3 | 50 | 40 |
| Mansour(133) | Lebanon | 2001 | Medical | Cigarettes | 4 | 171 | 27·7 (M) |
| Marin(135) | Argentina | 2006 | Mixed | Cigarettes | 3 | 1960 | 31·8 |
| Marochi(136) | Brazil | 2012 | Medical | Cigarettes | 2 | 32 | 3·2 |
| Maryana(138) | Malaysia | 2009 | Dental | Cigarettes | 2 | 720 | 13·8 |
| Mejia(142) | Argentina | 2005 | Medical | Cigarettes | 4.5 | 235 | 35·3 |
| Merrill(144) | Jordan | 2006 | Medical | Cigarettes | 4 | 251 | 19 |
| Nekhoroshev(155) | Russia | 2002 | Medical | Cigarettes | 4 | 1571 | 26 |
| Ocampo-Ocampo(159) | Mexico | 2000 | Mixed | Cigarettes | 1 | 659 | 28 |
| Okeke(160) | South Africa | 2009 | Medical Nursing Allied | Cigarettes | 5 | 94 276 156 | 11·7 8 16 |
| Oliveira(161) | Brazil | 2004 | Allied | Cigarettes | 3 | 194 | 17 |
| Ozturk(164) | Turkey | 2014 | Medical | Cigarettes | 4 | 151 | 15·9 |
| Peng(170) | China | 2012 | Medical | Cigarettes | 5 | 420 | 12·4 |
| Pourmahabadian(177) | Iran | 2002 | Mixed | Cigarettes | 2 | 124 | 38·7 |
| Pretto(179) | Brazil | 2012 | Mixed | Cigarettes | 2 | 340 | 5 |
| Prucha(182) | Dominican R | 2011 | Mixed | Tobacco | 4 | 107 | 3·7 |
| Retief(186) | South Africa | 2002 | Nursing | Cigarettes | 3 | 80 | 31·3 |
| Rodrigues(188) | Brazil | 2004 | Dental | Cigarettes | 5 | 234 (M) 212 (F) | 42 (M) 31·4(F) |
| Saavedra(190) | Peru | 2009 | Nursing | Cigarettes | 4 | 204 | 3 |
| Saglam(192) | Turkey | 2008 | Medical Nursing Mixed | Cigarettes | 2.5 | 169 260 691 | 18·7 34 36·9 |
| Sahebi(193) | Iran | 2006 | Mixed | Cigarettes | 2 | 1027 | 5·7 |
| Sanchez(198) | Ecuador | 2002 | Medical | Cigarettes | 5 | 509 (M) 170 (F) | 35·2 (M) 24·1(F) |
| Santos(201) | Brazil | 2011 | Medical | Cigarettes | 2 | 447 | 5·1 |
| Shi(206) | China | 2009 | Medical | Cigarettes | 4 | 256 (M) 211 (F) | 18·4 (M) 3·9(F) |
| Simonetti(208) | Brazil | 2005 | Nursing | Cigarettes | 2 | 235 | 13 |
| Smith(211) | China | 2005 | Mixed | Cigarettes | 4 | 315 | 10·5 |
| Smith(212) | China | 2008 | Medical | Cigarettes | 4.5 | 84 (M) 102 (F) | 46·7 (M) 5·3 (F) |
| Sonmez(213) | Turkey | 2014 | Medical Nursing | Cigarettes | 4.5 | 1182 1063 | 34·4 30·7 |
| Sousa(214) | Brazil | 2006 | Mixed | Cigarettes | 2 | 207 | 63 |
| Souza(215) | Brazil | 2011 | Nursing | Cigarettes | 1 | 50 | 12 |
| Suarez(217) | Cuba | 2007 | Nursing Dental Mixed | Cigarettes | 4 | 318 60 343 | 38·4 33·3 35·6 |
| Sun(218) | China | 2008 | Medical | Cigarettes | 3.5 | 732 (M) 923 (F) | 30·2 (M) 0·3 (F) |
| Torres(220) | Brazil | 2007 | Medical | Cigarettes | 4 | 1202 | 32·7 |
| Velasco-Contreras(231) | Mexico | 2009 | Medical Nursing Mixed | Tobacco | 3 | 3603 2581 19532 | 23·3 38·7 39·2 |
| Viegas(232) | Brazil | 2005 | Medical | Cigarettes | 5 | 830 | 7·2 |
| Villarroel(233) | Venezuela | 2007 | Dental | Cigarettes | 1 | 293 | 10·8 |
| Yan(236) | China | 2008 | Mixed | Cigarettes | 6 | 297 (M) 339 (F) | 43 (M) 1·2 (F) |
| Zhou(240) | China | 2007 | Medical | Cigarettes | 7 | 493 (M) 180 (F) | 35 (M) 3 (F) |
| Zylbersztejn(241) | Argentina | 2002 | Medical | Cigarettes | 2 | 783 | 28·6 |
| Zylbersztejn(242) | Argentina | 2004 | Medical | Cigarettes | 4 | 6497 | 30 |

HCW= healthcare workers; (M)=male; (F)=female.

**Table C. Summary of studies in high income countries (HIC)**

| **Author** | **Country** | **Year data collected** | **HCW Category** | **Type of tobacco use** | **Study quality score** | **Sample size** | **Tobacco use (%)** |
| --- | --- | --- | --- | --- | --- | --- | --- |
| Adamek(18) | Poland | 2011 | Nursing | Cigarettes | 3 | 73 (F) | 40 (F) |
| Akpanudo(20) | USA | 2005 | Medical | Cigarettes | 5 | 352 | 2·3 |
| Al-Arifi(22) | Saudi Arabia | 2003 | Pharmacy | Cigarettes | 6 | 261 | 19·9 |
| Al-Lawati(23) | Oman | 2001 | Medical | Cigarettes | 6 | 1191 | 11 |
| Arack(27) | England | 2007 | Mixed | Cigarettes | 4 | 160 | 19·5 |
| Awan(28) | Saudi Arabia | 2013 | Dental | Cigarettes | 4 | 130 | 33·8 |
| Azzopardi(30) | Malta | 2011 | Mixed | Cigarettes | 3 | 3600 | 27·1 (M) 24·8 (F) |
| Bazargan(35) | USA | 2008 | Medical | Cigarettes | 5.5 | 763 | 6 |
| Beaujouan(36) | France | 2001 | Medical | Cigarettes | 6 | 3428 | 22·7 |
| Beletsioti-Stika(37) | Greece | 2002 | Nursing | Cigarettes | 7 | 308 | 46 |
| Bello(38) | Chile | 2001 | Medical  Nursing  Mixed | Cigarettes | 4 | 1576  1326  20848 | 30·5  35·8  40·7 |
| Beltrán(39) | Spain | 2004 | Pharmacy | Cigarettes | 5 | 401 | 36·9 |
| Berkelmans(40) | Australia | 2007 | Nursing | Cigarettes | 7 | 58 (M) 965 (F) | 22·4 (M) 10·4 (F) |
| Boado(41) | Uruguay | 2011 | Medical | Cigarettes | 4 | 268 (M) 333 (F) | 46·3 (M) 38·7 (F) |
| Bolinder(42) | Sweden | 2001 | Medical | Cigarettes | 4 | 1094 | 6 |
| Bolinder(43) | Sweden | 2006 | Medical | Cigarettes  Snus | 6 | 1079 | 3  7 |
| Burgos Díez(45) | Spain | 2010 | Mixed | Cigarettes | 2 | 554 | 23 |
| Carel(48) | Israel | 2012 | Nursing | Cigarettes | 5 | 505 | 24·5 |
| Chang(52) | Taiwan | 2005 | Pharmacy | Cigarettes | 6 | 192 (M) 577 (F) | 18 (M) 0·5 (F) |
| Connolly(54) | New Zealand | 2012 | Nursing | Cigarettes | 2 | 104 | 30 |
| Cookson(55) | England | 2013 | Mixed | Cigarettes | 6 | 144 | 45 |
| Copertaro(56) | Italy | 2005 | Nursing | Cigarettes | 3 | 128 (M) 134 (F) | 32 (M) 23·1 (F) |
| Copertaro(57) | Italy | 2007 | Medical | Cigarettes | 3 | 128 (M) 286 (F) | 35 (M) 23 (F) |
| Cuesta(58) | Uruguay | 2004 | Medical Nursing | Cigarettes | 2 | 117 76 | 24 16 |
| De Col(60) | France | 2008 | Medical | Cigarettes | 6 | 332 | 18 |
| Díez Piña(64) | Spain | 2004 | Nursing  Allied | Cigarettes | 3 | 411 | 37·2  24·4 |
| Donchin(65) | Israel | 2000 | Mixed  Medical | Cigarettes | 2.5 | 345  63 | 18·6  12·7 |
| Downie(66) | Australia | 2013 | Allied | Cigarettes | 4 | 283 | 1 |
| Dwyer(67) | Australia | 2008 | Nursing | Cigarettes | 4.5 | 88 (M)  201 (F) | 21 (M)  13 (F) |
| Dziankowska-Zaborszczyk(68) | Poland | 2008 | Nursing | Cigarettes | 3 | 299 | 18 |
| Edwards(70) | New Zealand | 2006 | Nursing  Medical  Mixed | Cigarettes |  | 34896  9885  25083 | 14·2  3·6  12 |
| Estryn-Behar(71) | France | 2002 | Nursing | Cigarettes | 7 | 796 (M)  6184 (F) | 28·1 (M)  23·8 (F) |
| Fadhil(72) | Bahrain | 2005 | Medical | Cigarettes and water pipe | 5 | 120 | 24 |
| Faggianoi(73) | Italy | 2012 | Medical | Cigarettes | 6.5 | 1754 | 12·4 |
| Fathallah(75) | France | 2010 | Nursing | Cigarettes | 7 | 87 (M)  520 (F) | 32·3 (M)  29·8 (F) |
| Ferraz(76) | Portugal | 2009 | Nursing | Cigarettes | 3 | 105 | 16·2 |
| Fink(77) | USA | 2011 | Allied | Cigarettes | 3 | 263 | 1.1 |
| Fougere(78) | France | 2008 | Medical | Cigarettes | 3 | 606 | 25 |
| Freour(79) | France | 2009 | Medical | Cigarettes | 4 | 340 | 12·3 |
| Friis(80) | Denmark | 2000 | Nursing | Cigarettes | 6 | 22715 (F) | 23·4 (F) |
| Frisinghelli(81) | Italy | 2013 | Medical | Cigarettes | 4 | 451 (M)  159 (F) | 7 (M)  3 (F) |
| García de Albéñiz(82) | Spain | 2000 | Nursing | Cigarettes | 3 | 6711 | 42 |
| Gazdek(83) | Croatia | 2011 | Medical Nursing | Cigarettes | 2 | Not specified | 19·4 29·4 |
| Giotakis(85) | Greece | 2012 | Mixed | Cigarettes | 2 | 104 | 52 |
| Glavas(86) | Croatia | 2002 | Mixed | Cigarettes | 4 | 104 (M)  207 (F) | 36·5 (M)  35·7 (F) |
| Gowin(87) | Poland | 2008 | Medical | Cigarettes | 2 | 268 | 8·3 |
| Grempler(88) | Germany | 2007 | Mixed | Cigarettes | 3 | 410 | 34 |
| Hernández(93) | Spain | 2003 | Nursing | Cigarettes | 6 | 156 | 22 |
| Hilleshein(95) | Portugal | 2010 | Nursing | Cigarettes | 3 | 93 | 8·6 |
| Hjalmarson(96) | Sweden | 2003 | Allied | Cigarettes  Snus | 6.5 | 240 (M)  597 (F)  238 (M)  580 (F) | 20 (M)  15 (F)  23 (M)  4 (F) |
| Hung(98) | USA | 2013 | Medical | Medical | 3.5 | 1000 | 4 |
| Incorvaia(99) | Italy | 2007 | Mixed | Cigarettes | 2 | 383 | 25·8 |
| Jimenez-Ruiz(103) | Spain | 2014 | Medical  Nursing Mixed | Cigarettes | 7 | 416  196 612 | 11·1  11·4 11·7 |
| Kaiser(104) | Germany | 2004 | Medical | Cigarettes | 4 | 97 | 20 |
| Kaneita(105) | Japan | 2012  2008  2004  2000 | Medical | Cigarettes | 7 | 5854  3486  3633  3771 | 10·5  11·5  16·2  20·3 |
| Kara-Perz(107) | Poland | 2007 | Mixed | Cigarettes | 2 | 48 | 60·4 |
| Kazmi(108) | UK | 2008 | Mixed | Cigarettes | 3.5 | 195 | 24 |
| Kenfield(109) | USA | 2000 | Nursing | Cigarettes | 8 | 102635 (F) | 11·7 (F) |
| Khawaja(111) | USA | 2002 | Medical | Cigarettes | 8 | 21026 (M) | 2·8 (M) |
| Kim(113) | Korea | 2011 | Nursing | Cigarettes | 6 | 9989 (F) | 1·2 (F) |
| Kloppe(114) | Spain | 2000 | Medical | Cigarettes | 3 | 270 | 18·9 |
| Kobayashi(115) | Japan | 2010  2007  2005  2003  2001 | Mixed | Cigarettes | 2 | Not specified | 3·7  5·8  7·9  13·9  15·1 |
| Korzybski(116) | Poland | 2006 | Medical | Cigarettes | 5 | 134 (M)  272 (F) | 13 (M)  9 (F) |
| Kumbrija(119) | Croatia | 2007 | Medical  Nursing | Cigarettes | 4 | 125  89 | 26  44 |
| Kuo(120) | Taiwan | 2006 | Medical  Dental | Cigarettes | 3.5 | 491  124 | 21·6  15·3 |
| Lina(125) | Italy | 2011 | Medical | Cigarettes | 2 | 285 | 14 |
| Maksimovic(129) | Australia | 2012 | Mixed | Cigarettes | 4 | 85 | 50·6 |
| Malinauskiene(131) | Lithuania | 2005 | Nursing | Cigarettes | 6 | 748 (F) | 19·1 (F) |
| Mansoura(134) | Qatar | 2007 | Medical | Cigarettes | 5 | 85 (M)  66 (F) | 12·9 (M)  1·5 (F) |
| Martínez(137) | Spain | 2005 | Medical Nursing | Cigarettes | 4 | 3896 | 23.2 36 |
| Masia(139) | Italy | 2004 | Medical Nursing Allied | Cigarettes | 5 | 606 684 260 | 21·4 35·5 40·4 |
| Maurel-Donnarel(140) | France | 2008 | Nursing | Cigarettes | 6 | 84 (M)  370 (F) | 28·6 (M)  29·7 (F) |
| McKenna(141) | Northern Ireland, UK | 2000 | Nursing | Cigarettes | 5 | 1074 | 25·8 |
| Michalsen(145) | Germany | 2001 | Medical | Cigarettes | 5 | 296 | 47 |
| Mihalopoulos(146) | USA | 2007 | Medical | Cigarettes | 4 | 53 | 7 |
| Miyazaki(148) | Japan | 2001 | Nursing | Cigarettes | 7 | 49927 (F) | 17·2 (F) |
| Moreno(149) | Spain | 2014 | Mixed | Cigarettes | 1 | 37 | 28 |
| Moxham(151) | Australia | 2012 | Nursing | Cigarettes | 1 | 64 | 18·8 |
| Nappini(152) | Italy | 2014 | Nursing | Cigarettes | 4 | 42 (M)  205 (F) | 33·3 (M)  26·3 (F) |
| Negro(154) | Italy | 2006 | Mixed | Cigarettes | 2 | 2689 | 35 |
| O'Mahony(162) | Ireland | 2004 | Allied | Cigarettes | 1 | 174 | 28 |
| Osorio(163) | Chile | 2000 | Nursing | Cigarettes | 4 | 290 | 17·6 |
| Packer(165) | England, UK | 2013 | Medical | Cigarettes | 1 | 87 | 14 |
| Parkins(166) | Scotland, UK | 2011 | Mixed | Cigarettes | 4 | 192 | 7 |
| Pärna(167) | Estonia | 2002 | Medical | Cigarettes | 4.5 | 465 (M)  2203 (F) | 24·9 (M)  10·8 (F) |
| Pérez(171) | Spain | 2013 | Mixed | Cigarettes | 2 | 100 | 18 |
| Pericás(172) | Spain | 2005 | Nursing | Cigarettes | 6 | 376 | 26·7 |
| Perry(174) | Australia | 2011 | Nursing | Cigarettes | 5.5 | 382 | 18 |
| Poulsen(176) | Denmark | 2012 | Mixed | Cigarettes | 7 | 7305 | 36·6 |
| Pretti(178) | Italy | 2000 | Medical | Cigarettes | 6 | 3571 | 22·3 |
| Principe(180) | Italy | 2000 | Mixed | Cigarettes | 4 | 9843 | 33·3 |
| Proietti(181) | Italy | 2004 | Medical  Nursing | Cigarettes | 3 | 466  1002 | 27·7  36·3 |
| Pumpe(183) | Germany | 2002 | Mixed | Cigarettes | 4 | 510 | 26·9 |
| Rahman(184) | Australia | 2012 | Medical | Cigarettes | 4 | 1301 | 6·8 |
| Ravara(185) | Portugal | 2009 | Medical | Cigarettes | 1 | 226 (M)  381 (F) | 29·6 (M)  15·3 (F) |
| Rius(187) | Spain | 2010 | Medical | Cigarettes | 3 | 1098 | 14 |
| Rodríguez(189) | Spain | 2003 | Medical  Nursing | Cigarettes | 6 | 316  266 | 28  36·2 |
| Saeys(191) | Belgium | 2011 | Medical | Cigarettes | 5 | 359 (M)  267 (F) | 10 (M)  5 (F) |
| Saika(194) | Japan | 2004  2006 | Mixed | Cigarettes | 3 | 261  398 | 6·1  8·5 |
| Sainz(195) | Spain | 2006 | Mixed | Cigarettes | 5 | 111 (M)  283 (F) | 27·9 (M)  35·7 (F) |
| San Pedro(197) | Spain | 2005 | Medical | Cigarettes | 2 | 106 | 35·9 |
| Santa-María(199) | Spain | 2004 | Medical  Mixed | Cigarettes | 4 | 157  220 | 29·7  45 |
| Santander(200) | Chile | 2009 | Medical | Cigarettes | 3 | 96 (M)  67 (F) | 29·2 (M)  29·9 (F) |
| Sarna(202) | Czech Rep | 2014 | Nursing | Cigarettes | 4 | 157 (F) | 29·5 (F) |
| Sarna(203) | USA | 2007 | Nursing | Cigarettes | 8 | 2566 (F) | 12·1 (F) |
| Sarna(244) | USA | 2003 2003 2003 2003 2003  2007 2007 2007 2007 2007 | Medical Nursing Phramacy Dental Allied  Medical Nursing Phramacy Dental Allied | Cigarettes | 6.5 | 612 2828 203 148 301  600 2762 192 122 300 | 2·2 13·7 7·8 4 12·3  2·3 13·2 3·6 4 11·7 |
| Sarna(245) | USA | 2011 2011 2011 2011 | Medical Nursing Phramacy Allied | Cigarettes | 6.5 | 577 1947 225 300 | 2 11·9 3·5 8·4 |
| Schulz(204) | Germany | 2003 | Medical | Cigarettes | 3 | 329 | 41 |
| Shkedy(207) | Israel | 2012 | Medical | Cigarettes | 3 | 53 (M)  37 (F) | 18·9 (M)  13·5 (F) |
| Siqués(209) | Chile | 2001 | Mixed | Cigarettes | 4 | 176 | 37 |
| Slater(210) | Northern Ireland, UK | 2005 | Nursing | Cigarettes | 5 | 86 (M)  988 (F) | 38·1 (M)  24·6 (F) |
| Stubbs(216) | Ireland | 2003 | Medical  Nursing  Mixed | Cigarettes | 3 | 19  476  599 | 0  26·1  22 |
| Tong(219) | USA | 2003 | Medical  Nursing  Dental  Pharmacy  Allied | Cigarettes | 7 | 1245  388  391  403  377 | 3·5  13·1  5·8  4·4  5·3 |
| Tountas(221) | Greece | 2000 | Mixed | Cigarettes | 2 | 101 (M)  161 (F) | 46 (M)  43 (F) |
| Tselebis(224) | Greece | 2000 | Nursing | Cigarettes | 2 | 114 (F) | 46 (F) |
| Uallachain(225) | Ireland | 2003 | Medical | Cigarettes | 4 | 100 | 8 |
| Underner(226) | France | 2002 | Medical | Cigarettes | 5 | 189 (M)  68 (F) | 27·6 (M)  20·6 (F) |
| Uruena(227) | Spain | 2001 | Medical  Nursing | Cigarettes | 3 | 90  121 | 19  26 |
| Valverde(229) | Spain | 2011 | Medical | Cigarettes | 5 | 215 | 19 |
| Vilarroel(233) | Spain  Italy | 2007 | Dental | Cigarettes | 1 | 90  110 | 51  20 |
| Virtanen(234) | Finland | 2004 | Mixed | Cigarettes | 3.5 | 8003 | 22·7 |
| Vitzthum(235) | Germany | 2010 | Mixed | Cigarettes | 5 | 1835 | 20 |
| Zapka(239) | USA | 2005 | Nursing | Cigarettes | 4.5 | 194 | 5·9 |
| Zysnarska(243) | Poland | 2008 | Nursing | Cigarettes | 2 | 118 | 27 |

HCW= healthcare workers; (M)=male; (F)=female.
