# Supplementary material for: Prevalence of tobacco use in healthcare workers: A systematic review and meta-analysis
Source: PLoS One. 2019 Jul 25;14(7):e0220168. doi: 10.1371/journal.pone.0220168 (PMC6657871; doi:10.1371/journal.pone.0220168)
Supplement: S5 Appendix — (DOCX) [file pone.0220168.s005.docx]

# S5 Appendix. Country level comparison of prevalence of tobacco use in HCW and the general population.

**Table A. Pooled prevalence of tobacco use in male HCW compared with males in the general population**

| **Country** | **Country Income Level** | **Year Data Mean** | **HCW Males (Mean) %** | **Year** | **Males general population, %** | **Difference, %** | **Prevalence Comparison** |
| --- | --- | --- | --- | --- | --- | --- | --- |
| Bahrain | High | 2005 | 26.3 | 2015 | 48.8 | -22.5 | lower in HCW |
| Israel | High | 2006 | 18.9 | 2015 | 41.2 | -22.3 | lower in HCW |
| Ireland | High | 2003 | 0.5 | 2015 | 22.4 | -21.9 | lower in HCW |
| Lithuania | High | 2005 | 17.3 | 2015 | 38.1 | -20.8 | lower in HCW |
| Poland | High | 2008 | 13 | 2015 | 32.4 | -19.4 | lower in HCW |
| USA | High | 2005 | 2.8 | 2015 | 19.5 | -16.7 | lower in HCW |
| Belgium | High | 2011 | 10 | 2015 | 26.5 | -16.5 | lower in HCW |
| Estonia | High | 2002 | 24.9 | 2015 | 41.2 | -16.3 | lower in HCW |
| Japan | High | 2005 | 19 | 2015 | 33.7 | -14.7 | lower in HCW |
| Chile | High | 2002 | 29.2 | 2015 | 40 | -10.8 | lower in HCW |
| Oman | High | 2001 | 16.4 | 2015 | 21 | -4.6 | lower in HCW |
| Spain | High | 2006 | 27.1 | 2015 | 31.3 | -4.2 | lower in HCW |
| Croatia | High | 2008 | 36.5 | 2015 | 39.4 | -2.9 | lower in HCW |
| Malta | High | 2011 | 27.1 | 2015 | 29.7 | -2.6 | lower in HCW |
| Sweden | High | 2004 | 18 | 2015 | 20.4 | -2.4 | lower in HCW |
| France | High | 2006 | 27.8 | 2015 | 29.8 | -2 | lower in HCW |
| Portugal | High | 2009 | 29.6 | 2015 | 31.5 | -1.9 | lower in HCW |
| Greece | High | 2004 | 50.8 | 2015 | 52.6 | -1.8 | lower in HCW |
| Australia | High | 2011 | 18.3 | 2015 | 16.7 | 1.6 | higher in HCW |
| Italy | High | 2006 | 30.6 | 2015 | 28.3 | 2.3 | higher in HCW |
| Uruguay | High | 2006 | 46.3 | 2015 | 26.7 | 19.6 | higher in HCW |
| Qatar | High | 2007 | 12.9 | 2015 | no data | n/a | n/a |
| Russia | UM | 2002 | 33 | 2015 | 59 | -26 | lower in HCW |
| Bosnia&Herzegovina | UM | 2002 | 33.3 | 2015 | 47.2 | -13.9 | lower in HCW |
| China | UM | 2008 | 37.6 | 2015 | 47.6 | -10 | lower in HCW |
| Lebanon | UM | 2001 | 36.2 | 2015 | 45.4 | -9.2 | lower in HCW |
| Argentina | UM | 2006 | 29.4 | 2015 | 29.5 | -0.1 | lower in HCW |
| Mexico | UM | 2008 | 23.7 | 2015 | 20.8 | 2.9 | higher in HCW |
| Turkey | UM | 2008 | 42.5 | 2015 | 39.5 | 3 | higher in HCW |
| Brazil | UM | 2008 | 28 | 2015 | 19.3 | 8.7 | higher in HCW |
| Iran | UM | 2007 | 37.4 | 2015 | 21.5 | 15.9 | higher in HCW |
| Ecuador | UM | 2002 | 35.2 | 2015 | 14 | 21.2 | higher in HCW |
| Peru | UM | 2009 | 67.2 | 2015 | no data | n/a | n/a |
| Venezuela | UM | 2007 | 38.7 | 2015 | no data | n/a | n/a |
| Indonesia | LM | 2003 | 22 | 2015 | 76.2 | -54.2 | lower in HCW |
| Armenia | LM | 2007 | 36.5 | 2015 | 52.3 | -15.8 | lower in HCW |
| India | LM | 2011 | 7.3 | 2015 | 20.4 | -13.1 | lower in HCW |
| Pakistan | LM | 2009 | 56.2 | 2015 | 41.9 | 14.3 | higher in HCW |
| Syria | LM | 2011 | 35 | 2015 | no data | n/a | n/a |
| Tunisia | LM | 2008 | 53 | 2015 | no data | n/a | n/a |
| Nepal | Low | 2009 | 24 | 2015 | 37.1 | -13.1 | lower in HCW |

**Table B. Pooled prevalence of tobacco use in female HCW compared with females in the general population**

| **Country** | **WB Income Level** | **Year Data Mean** | **HCW Females (Mean),%** | **Year** | **Females general population %** | **Difference , %** | **Prevalence Comparison** |
| --- | --- | --- | --- | --- | --- | --- | --- |
| Uruguay | High | 2006 | 38.7 | 2015 | 19.4 | 19.3 | higher in HCW |
| Greece | High | 2004 | 46.1 | 2015 | 32.7 | 13.4 | higher in HCW |
| Spain | High | 2006 | 36.4 | 2015 | 27.1 | 9.3 | higher in HCW |
| Denmark | High | 2006 | 23.4 | 2015 | 16.4 | 7 | higher in HCW |
| Malta | High | 2011 | 24.8 | 2015 | 20.2 | 4.6 | higher in HCW |
| Italy | High | 2006 | 23.7 | 2015 | 19.7 | 4 | higher in HCW |
| Australia | High | 2011 | 16.3 | 2015 | 13.1 | 3.2 | higher in HCW |
| Croatia | High | 2008 | 35.7 | 2015 | 33.5 | 2.2 | higher in HCW |
| Portugal | High | 2009 | 15.3 | 2015 | 13.7 | 1.6 | higher in HCW |
| Czech Republic | High | 2014 | 29.5 | 2015 | 29 | 0.5 | higher in HCW |
| France | High | 2006 | 25.4 | 2015 | 25.6 | -0.2 | lower in HCW |
| Oman | High | 2001 | 0.3 | 2015 | 1 | -0.7 | lower in HCW |
| Lithuania | High | 2005 | 19.1 | 2015 | 22.2 | -3.1 | lower in HCW |
| USA | High | 2005 | 11.9 | 2015 | 15 | -3.1 | lower in HCW |
| Japan | High | 2005 | 7.4 | 2015 | 10.6 | -3.2 | lower in HCW |
| Israel | High | 2006 | 13.5 | 2015 | 19.3 | -5.8 | lower in HCW |
| Chile | High | 2002 | 29.9 | 2015 | 36 | -6.1 | lower in HCW |
| Ireland | High | 2003 | 10 | 2015 | 21.9 | -11.9 | lower in HCW |
| Sweden | High | 2004 | 7.3 | 2015 | 20.8 | -13.5 | lower in HCW |
| Estonia | High | 2002 | 10.8 | 2015 | 24.9 | -14.1 | lower in HCW |
| Poland | High | 2008 | 9 | 2015 | 23.7 | -14.7 | lower in HCW |
| Belgium | High | 2011 | 5 | 2015 | 20 | -15 | lower in HCW |
| Korea | High | 2011 | 1.2 | 2015 | no data | n/a | n/a |
| Qatar | High | 2007 | 1.5 | 2015 | no data | n/a | n/a |
| Ecuador | UM | 2002 | 24.1 | 2015 | 3.3 | 20.8 | higher in HCW |
| Cuba | UM | 2007 | 38.4 | 2015 | 17.8 | 20.6 | higher in HCW |
| Jordan | UM | 2006 | 30 | 2015 | 10.7 | 19.3 | higher in HCW |
| Bosnia&Herzegovina | UM | 2002 | 48 | 2015 | 30 | 18 | higher in HCW |
| Turkey | UM | 2008 | 26.7 | 2015 | 12.4 | 14.3 | higher in HCW |
| Argentina | UM | 2006 | 31 | 2015 | 18.4 | 12.6 | higher in HCW |
| Brazil | UM | 2008 | 19.9 | 2015 | 11.3 | 8.6 | higher in HCW |
| Mexico | UM | 2008 | 12.6 | 2015 | 6.6 | 6 | higher in HCW |
| South Africa | UM | 2007 | 8 | 2015 | 6.5 | 1.5 | higher in HCW |
| Iran | UM | 2007 | 1.2 | 2015 | 0.7 | 0.5 | higher in HCW |
| Russia | UM | 2002 | 21.1 | 2015 | 22.8 | -1.7 | lower in HCW |
| Jamaica | UM | 2004 | 4 | 2015 | 5.9 | -1.9 | lower in HCW |
| Lebanon | UM | 2001 | 17.3 | 2015 | 31 | -13.7 | lower in HCW |
| Armenia | LM | 2007 | 14.5 | 2015 | 1.5 | 13 | higher in HCW |
| Pakistan | LM | 2009 | 10 | 2015 | 3 | 7 | higher in HCW |
| India | LM | 2011 | 0.5 | 2015 | 1.9 | -1.4 | lower in HCW |
| Indonesia | LM | 2003 | 0.5 | 2015 | 3.6 | -3.1 | lower in HCW |
| Syria | LM | 2011 | 4 | 2015 | no data | n/a | n/a |
| Tunisia | LM | 2008 | 10 | 2015 | no data | n/a | n/a |
| Nepal | Low | 2009 | 1.8 | 2015 | 11.1 | -9.3 | lower in HCW |
