# Supplementary material for: Prevalence of tobacco use in healthcare workers: A systematic review and meta-analysis
Source: PLoS One. 2019 Jul 25;14(7):e0220168. doi: 10.1371/journal.pone.0220168 (PMC6657871; doi:10.1371/journal.pone.0220168)
Supplement: S6 Appendix — (DOC) [file pone.0220168.s006.doc]

# S6 Appendix. Meta-analyses.

**Figure A. Pooled prevalence of tobacco use in HCW in HIC by year of data collection (2000-2005), % (95 % CI)[[1]](#footnote-2)**

HCW= healthcare workers

**Figure B. Pooled prevalence of tobacco use in HCW in HIC by year of data collection (2006-2010), % (95 % CI)**[[2]](#footnote-3)

HCW= healthcare workers

**Figure C. Pooled prevalence of tobacco use in HCW in HIC by year of data collection (2011-2015), % (95 % CI)[[3]](#footnote-4)**

HCW= healthcare workers

**Figure D.** Pooled prevalence of tobacco use in HCW in UMIC by year of data collection, % (95 % CI)**[[4]](#footnote-5)**

HCW= healthcare workers

**Figure E. Pooled prevalence of tobacco use in HCW in LMLIC by year of data collection, % (95 % CI)[[5]](#footnote-6)**

HCW= healthcare workers

# Figure F. Pooled prevalence of tobacco use in male HCW by income level % (95 % CI)[[6]](#footnote-7)

HCW= healthcare workers

# Figure G. Pooled prevalence of tobacco use in female HCW by income level % (95 % CI)[[7]](#footnote-8)

HCW= healthcare workers

**Figure H. Pooled prevalence of tobacco use in medical HCW in HIC % (95 % CI)[[8]](#footnote-9)**

HCW= healthcare workers

**Figure I. Pooled prevalence of tobacco use in medical HCW in UMIC % (95 % CI)[[9]](#footnote-10)**

HCW= healthcare workers

**Figure J. Pooled prevalence of tobacco use in medical HCW in LMLIC % (95 % CI)[[10]](#footnote-11)**

HCW= healthcare workers

**Figure K. Pooled prevalence of tobacco use in nursing HCW by income level % (95 % CI)[[11]](#footnote-12)**

HCW= healthcare workers

**Figure L. Pooled prevalence of tobacco use in dental HCW by income level % (95 % CI)[[12]](#footnote-13)**

HCW= healthcare workers

**Figure M. Pooled prevalence of tobacco use in pharmacy HCW by income level % (95 % CI)[[13]](#footnote-14)**

HCW= healthcare workers

**Figure N.** **Pooled prevalence of tobacco use in allied HCW by income level % (95 % CI)[[14]](#footnote-15)**

HCW= healthcare workers

**Figure O. Pooled prevalence of tobacco use in mixed HCW by income level % (95 % CI)[[15]](#footnote-16)**

HCW= healthcare workers

**Figure P. Pooled prevalence of tobacco use male medical HCW by income level % (95 % CI)[[16]](#footnote-17)**

HCW= healthcare workers

**Figure Q. Pooled prevalence of tobacco use female medical HCW by income level % (95 % CI)[[17]](#footnote-18)**

HCW= healthcare workers

**Figure R. Pooled prevalence of tobacco use male nursing HCW by income level % (95 % CI)[[18]](#footnote-19)**

HCW= healthcare workers

**Figure S. Pooled prevalence of tobacco use female nursing HCW by income level % (95 % CI)[[19]](#footnote-20)**

HCW= healthcare workers

1. Study score range 1 to 8. [↑](#footnote-ref-2)
2. Study score range 1 to 8. [↑](#footnote-ref-3)
3. Study score range 1 to 7. [↑](#footnote-ref-4)
4. Study score range 1to 7. [↑](#footnote-ref-5)
5. Study score range 2 to 6. [↑](#footnote-ref-6)
6. Study score range 1 to 7. [↑](#footnote-ref-7)
7. Study score range 1 to 8. [↑](#footnote-ref-8)
8. Study score range 1 to 7. [↑](#footnote-ref-9)
9. Study score range 1 to 7. [↑](#footnote-ref-10)
10. Study score range 2 to 6. [↑](#footnote-ref-11)
11. Study score range 1 to 8. [↑](#footnote-ref-12)
12. Study score range 1 to 7. [↑](#footnote-ref-13)
13. Study score range 4.5 to 7. [↑](#footnote-ref-14)
14. Study score range 1 to 7. [↑](#footnote-ref-15)
15. Study score range 1 to 7. [↑](#footnote-ref-16)
16. Study score range 1 to 7. [↑](#footnote-ref-17)
17. Study score range 1 to 7. [↑](#footnote-ref-18)
18. Study score range 3 to 7. [↑](#footnote-ref-19)
19. Study score range 2 to 8. [↑](#footnote-ref-20)
